# Exiting the captaverse: Digital resistance and its limits pre and post the Covid-19 pandemic

**DOI:** 10.1177/17488958231184695

**Published:** 2023-07-14

**Authors:** Janos Mark Szakolczai

**Affiliations:** University of Glasgow, UK

**Keywords:** Big Brother, bigdata, capta, Covid-19, covidiots, cybercrime, Deleuze, desistance, digital harm, e-health, FOMO, obfuscation, onlife, panopticon, surveillance

## Abstract

The term ‘data’, ubiquitous in the Digital Age, etymologically refers to a piece of information ‘given’ (datum). In this article, I argue that the term ‘capta’ would be more accurate, since information is often taken from us. Capturing information replicates normative elements of abuse, surveillance, control and harm becoming central and problematic within the emergence of the ‘onlife’. I illustrate my argument via an ethnomethodological consideration of my attempt to resist the unwilling capture of personal information. Since 2016, I have engaged in what I call an ‘offlife’ existence, phasing out all devices and platforms that covertly capture personal data. However, my experiment has proven problematic, impractical and has even been perceived as being anti-social, especially in light of the Covid-19 pandemic. In the conclusion of this article, I consider the problematics of engaging in some form of resistance to data collection.

## Data-as-given

The word ‘Data’ originates from Latin: it is a datum; something given. Thus, a piece of something, namely, a piece of information, ‘handed’ consensually out. The use of ‘data’ is a recurring problem of our Internet engagement. ‘Data’ are taken and kept from users – analysed and monitored, on a regular and spiralling and “liquid” basis (Bauman and Lyon, 2013). This metrical gathering, storing and recording (Beer, 2016) of users’ digital activity, including clicks, swipes, likes, purchases, movements, behaviours and interests (Christl and Spiekermann, 2016; Lyon, 2018), and generalised use of biometrics (Pugliese, 2012), occurs in a ubiquitous and unsanctioned manner (Nissenbaum, 2015). This leads to the extreme offering of an open view into our ‘soul’, in the poignant words of G.T. Marx (2016), especially recalling a ‘totality’ of elements of harm, surveillance, monitoring and control (Giglioli, 2019). The currency of the ‘cyberage’ has been connected within the ‘big picture’ of harm (Redden et al., 2020), intensifying among others, elements of cyberstalking, flashing (McGlynn and Johnson, 2021), aggression (Marganski and Melander, 2018), doxing, trolling (Lavorgna, 2021) and toxic social surveillance (Trottier, 2019). This is particularly significant if we acknowledge the rhizomatic means of control (Deleuze, 1992), and the surveillance assemblage it constitutes (Haggerty and Ericson, 2000), leading to the quintessential ‘dragnet’ environment (Angwin, 2014) academics and researchers have been investigating for the last decades. From the perspective of digital harms (Wood, 2022), cyber harms have become an encompassing reality (Lavorgna, 2021), limiting any resistance, and taking over what Luciano Floridi (2015) considered an ‘onlife’ engagement (online + life). The theoretical bases of this article are the considerations offered about aspects of control in the digital age by Philip E. Agre and Gilles Deleuze. In particular, Agre (1994) saw the elements of ‘Captivating Surveillance’ as a growing aspect of our digital engagement and relation with technology. Deleuze (1992) perceived the novel and encompassing elements of control that evolved from Foucault’s (2008 (1978)) disciplining societies. I explore the ideas of Deleuze and Agre not only due to their theoretical relevance but (metaphorically) also as real-life responses and dissent to the englobing scenario they saw before them. Deleuze incidentally opted out of his dystopic Society of Control ending his life in 1995. Agre chose to go ‘off the grid’, and literally disappeared in 2009 (Pescovitz, 2009). My article discusses my ethnographical attempt to engage with an alternative, from 2016 through (most of) the Covid-19 pandemic. Having grown up in the digital revolutions of the late 2000s, I literally dived into all major social networks and interacted enthusiastically with all the latest mobile technology & smart media novelties. Though curious, my involvement was never without criticism, and over time my concerns grew over the potential, the manoeuvres and the implications these tools have on users. My scholarly objective was to allow an alternative sense of ‘agency’, as opposed to the fatalism of harmful data collection dynamics. Ultimately, I came to explore the idea of something not quite right in the implications of ‘handing-out’ our ‘datum’, and what, other than privacy concerns, is the harm involved.

The first step to do so was seeking a so-called ‘offlife’ engagement as to resist the rhizomatic formation of abusive data collection (Haggerty and Ericson, 2000). This involved specifically a social media suicide, self-limited Internet involvement and full-scale disengagement from smart devices. My objective was not simply to produce research data, but to become engaged in a novel, experimental hybrid ethnomethodological practice (Przybylski, 2020) that would be less problematic for me as a person and a user of the onlife. This aspect is central and significant today more than ever, in light of the setting of new scenarios for further data collection within the metaverse (Beer, 2022), and the means implied for the gestation of the Covid-19 pandemic. In both instances, potential harm appears underplayed. That ‘privacy is dead’ is a recurring mantra that has been recalled to us repeatedly, among others by Zuckerberg, who underlined decades ago how privacy is no longer a social norm (Johnson, 2010). Though data collection is still to date perceived as controversial and problematic, at least from a privacy standpoint, its resistance is just as well quite limited and ignored (Brunton and Nissenbaum, 2015). Refusing the capturing of information becomes not simply a privacy issue (Lavorgna, 2021), but a question of resistance (see Ferrell, 2022), dissent (Selmini and Chiaramonte, 2022) and even, more specifically, a quest into modelling a subjectivity resistant to capta.^1^ In this context, the connection of data and harm, including the cybercriminal and toxic elements of our constantly connected devices, becomes a focal element of our onlife experience. In contrast, my experimental offlife ecology proposes a perhaps utopian environment where users are more in control of their information and their data. The requirement is for our data – even when published voluntarily – to free itself from a *capta* establishment that perpetuates the conditions of having something taken and used covertly. Such an approach appears central in creating awareness and at the same time a guideline for resistance to avoid automatic flagging of those who are not integrated and limit the control of the biopolitical apparatus (Agamben, 2009), or, to put it simply, to permit some sort of alternative that does not imply some repercussion or make one look like a criminal (Vertesi, 2015).

## Captaveillance

Digital data are trivially understood as the exchange we offer, almost reciprocally, for ‘free navigation’. This gratuity is meaningless in a neoliberal milieu, being rather the compromise of a ‘no-free-gift’ (Douglas, 1950 (1990)) condition we have become accustomed to within our society. When users divulge and ‘give away’ their information, it appears as something ‘out there for [anyone] to use’ (Marx, 1998: 178): the idea of consent or choice within the online realm is ‘disingenuous to the extreme’ (Marx, 1998: 178). While we are offered consent forms when navigating the web, at least according to various Western regulations, what really takes place in the background of our digital existence is out of our reach or knowledge. Such argument is led forward by Byoung Chul-Hal in describing an aperspectival panopticon system, underlining its ‘omnipotence of the despotic gaze . . . of everyone from everywhere, which anyone can perform’ (Han, 2012: 45). As shown by yet another significant Facebook/Meta leak, data collection becomes not only as replete and boundless as ever but also impossible to keep track of (Biddle, 2022).

This falls short of Deleuze’s conceptualisation of a ‘surveillance assemblage’ (Haggerty and Ericson, 2000), a prison-like yet discrete system where we are granted access to places and spaces only by constantly showing some electronic identification. ‘Individuals have become “dividuals” and masses, samples, data, markets, or “banks”’ (Deleuze, 1992: 5); non-abidance only leads to further marginalisation and exclusion. Internet critic Geert Lovink (2019) and scholars like David Lyon (2018, 2022), noticed how the devices we carry perpetually define us, sort us and enlist us, with potentially demeaning means. For Deleuze, who took forward Foucault’s theories on the centrality of discipline within the notion of blind surveillance, the Societies of Control are mixed within an automatised system – an assemblage where surveillance grows into a truly rhizomatic apparatus, conceived as operating ‘by variation, expansion, conquest, capture, offshoots’ (Deleuze and Guattari, 1987: 21).

Such taking rather than receiving was a central concern of Philip Agre already 1994, when discussing the nature and problematics of ‘Captivating Surveillance’. The action of ‘capturing’ is for Agre an ‘upgrade’ to the Big Brother–like surveillance milieu. In detail, ‘The capture model, like the surveillance model, is a metaphor system and not a literal description’ (Agre, 1994: 107). The question of tracking in Agre’s (1994) work is connected to automatism and Taylorism, addressing the twin imperatives of efficiency and control in the same fashion: ‘by legislating the precise sequence of actions in advance’ (Agre, 1994: 117).

Especially since the onlife engagement (Floridi, 2015), data are not simply a piece of information; it is the unity ‘used to provide some sort of measure of the world’ (Beer, 2016: 3). Data are the oil that lubricates the engagement, the advertisement, the tailoring and the functioning of the digital experience. These pieces of information are taken and kept from users – analysed, gathered and stored, on a regular and spiralling basis (Pasquale, 2015). The word ‘data’ is indeed perhaps too kind, if not simply misleading, because it gives a different light on what takes place, how and why. I suggest that the word ‘datum’ be changed to ‘capta’ in situations where harmful and secretive collection is taking place. This will better convey the idea of something being taken, rather than simply given.^2^ These considerations over *data* and *capta* recur in other disciplines, such as archaeological research (Chippindale, 2000) and social sciences (Lanigan, 1994). The discussion is surprisingly stale and has a somewhat anachronistic reckoning. Capta appears as such not only the currency, but the coercive power of the systems built around us. It is a surveillance that takes all; it is purposeless if not to capture, that is, featuring a captaveillance dynamic. Capturing, as we will now discuss in detail, becomes a truly ‘agnostic’ (Lanigan, 1994: 116), and thus limitless stance. It shapes lifestyles and becomes central in the metrical form of power: ‘the means by which our lives are captured, but in which that information is protected by commercial interests’ (Beer, 2016: 108).

## Captivating devices

Capturing information is a replete and recurring scenario. It takes place both in passive and in active terms. *Passive* captaveillance is evident with devices such as smartphones. Smartphones offer the perfect device and medium for this ‘captivating’ condition. It induces and partly seduces the user, thanks to the design of its features, inducing a specific ‘data-producing’ life. As noted by Giglioli (2019), this form of ‘indirect’ surveillance involves us, without ever actually seeming to ‘touch’ us. The smart tools are designed to produce and store pieces of information in what Siva Vaidhyanathan (2012) noted as a ‘cryptopticon’, drawing towards the ‘cryptic, hidden, scrambled and mysterious’ (Vaidhyanathan, 2018: 67) monitoring reality, where ‘one can never be sure who is watching who and for what purpose’ (Vaidhyanathan, 2018: 67). A smartphone, for example, is constantly in the user’s pocket, functioning both when capturing signal or whether in ‘aeroplane mode’: it collects all sorts of information about users’ whereabouts and usage with the use of sensors (GPS, barometers, altimeters) and usage-monitoring (screen time, clicks, interests, communication) offers trivial functions for surveillance and monitoring, containing our most private information (including eHealth, more later). The design of our devices thus is only increasingly replicating the black-box creeping functions described by Frank Pasquale (2015), along with the crypto-elements of control and monitoring of everyone and everything (Herrero et al., 2021). The smartphone replicates the capturing of information, and does so endlessly, with new and new systems and upgrades. It becomes an agnostic scenario that users struggle to conceive, comprehend and limit – a point that is rendered even more clear with Dave Beer’s consideration of algorithms forming a new social power that controls and monitors our web content (Beer, 2017).

From an *active* perspective, captaveillance involves users in ‘taking’ pictures, filming and audio recording; the ‘capturing’ of what is around is done with evident nonchalance, regular conduct that anyone with a smartphone can trivially, almost unconsciously engage with – showing very limited and blasé concern towards privacy violations (Giglioli, 2019). As Vaidhyanathan (2012) had noted already a decade ago, none of this was possible without smartphones. Nowadays, in the aftermath of the Covid-19 pandemic, we are more than ever limited within our connected yet detached compound.

From a criminological perspective, any Internet-connected device, especially due to its ‘smart’ functions, allows an active endless replication of all the elements of cyber harm: from stalking through doxing to doing surveillance – providing instances of capta collection. Cyber criminality has become an accepted if not trivial form of crime and criminality (Yar, 2005). What is permitted, and to what we have ‘given’ permission, loses its grasp in virtual scenarios. Cyberstalking as a form of captaveillance is significant, as an ‘invasive form of partner monitoring’ (Marcum et al., 2017: 375) can take place in apparent ‘friendly’ social environments, particularly aided by social networking platforms that become per se social surveillance websites (Tokunaga, 2011). Such episodes take place on university campuses (Marcum et al., 2017) as well as within internal communications of co-workers (Lowry et al., 2016). It takes place globally and in an all-aged (Horst, 2020) and pluri-gendered (Winkelman et al., 2015) reality. Stalking and bullying are particularly connected in this scenario, due to the insistence of offences (Forssell, 2016; Lavorgna, 2014; Shariff, 2014). Such dynamics show evidence of ‘more psychosocial and emotional damage than traditional offline physical bullying because of the increased volume, scale, scope, and number of witnesses’ (Gillespie, 2009, cited in Lowry et al., 2016: 963).

## Going ‘offlife’

By the mid-2010s, I contemplated how to detach myself, from the abovementioned harmful dynamics, without paying the price of a secluded life: a user of the onlife existence but at the same time an *offlife* participant. This was important for me both as a citizen and as a social scientist. I conceived the ‘offlife’ as a way of integrating Floridi’s (2015) neologism ‘onlife’. To be ‘offlife’ for me did not simply mean going ‘offline’, or ‘off-the-grid’ (Angwin, 2014). Instead, it consisted in systematically limiting the number of online sources, platforms and devices – in the likes of the notorious experiments by Vertesi (2015) who attempted to reduce the online visibility of her pregnancy. However, I did not wish to disappear, if at all possible (Haggerty and Ericson, 2000: 619), nor strenuously confuse or mislead governments and corporations, but rather limit my ‘capta’ while engaging in a healthy social, cultural, intellectual and productive life. As the onlife existence involving smartphones, social media, networks, online maps, interactive apps, tracking devices, and freemium games surround us every day in a regular and complementary fashion, even a simple glimpse of the surroundings proved a significant source of study.

Building up from cultural criminology, my approach was much in tune with the ethnomethodological methods (Garfinkel, 1967), trying to identify the ‘everyday life world’ of onlife users and conceptualise ‘drift’ methods of engaging with their experiences (Ferrell, 2018). I offer a reflexive sociological and criminological consideration of the harms involved in captaveillance, faithful to ‘the myriad forms of resistance and the repressive nature of acquiescence’ (Ferrell et al., 2008: 205). All related considerations have rested solely on my lived experience – which becomes a central disengaged offlife element ‘within’ the onlife environment.

The initial difficulty involved in ‘not-playing-by-the-rules’ became evident when choosing the strictest-possible privacy-focused options on my browser. The result is one of not being allowed a proper navigation experience: access was limited or practically impossible on most websites. This, it seems, recalls the conditions as suggested by Deleuze (1992): Without the proper ‘conduct’ and acceptance of the ‘rules’ and conditions offered by the platforms, access is not granted.

To continue navigating freely, I instead followed the lead of obfuscating methods (Brunton and Nissenbaum, 2015). The approach of these scholars and computer engineers is a particularly helpful attempt to scramble users’ data both online and offline. Using VPN software, privacy-friendly search tools, multi-layer encryption browsers and disposable emails helped to significantly reduce my clickstream, metadata and logs, thus avoiding or at least reducing to a minimum the ‘very specific and personal narrative’ (Brunton and Nissenbaum, 2015: 54). Such tools could have been well integrated with other platforms such as Duck Duck Go browsers, or even more efficiently (though somewhat lagging the browsing experience) via Tor and Dark Web that allow strict minimisation of captaveillance technologies. Also, I installed specific ‘add-on’ extensions on my everyday Internet navigation browsers, limiting tracking, disagreeing automatically to cookie collection and scrambling my very ‘data’, confusing it with non-pertinent information.^3^

However, obfuscating solutions and platform extensions do not limit the practice of captaveillance. Not that they fail in doing so, rather they are designed differently. Also, there is no evidence whether the corporation cannot very well, and precisely, recognise what content is ‘bot’ produced, and what is still perfectly identifiable and targeted to the ad personam user.^4^ Moreover, many of the very add-ons and alternative browsers rendered navigation limited, on some websites impossible.

It became clear that if I cannot access social media and most websites on my terms, it made rather more sense to do without them altogether. I thus eventually decided to take a more ‘radical’ stance, the effects of which I will now illustrate seeking to avoid the occurrence of capta with the aid of four onlife contexts. I began by deleting all my social media accounts, limiting myself to the use of smart devices, engaging only with analogical audio-video devices and generally reducing my digital footprint.

### Social media

Indeed, as the limitation set by these self-imposed ‘terms and conditions’ made all navigation to prime social media services impossible, I decided in June 2016 to opt out and delete all my social media accounts.

Initial reactions to my disengagement led, to say the very least, to annoyance among my peers and the suggestion that I was ‘missing out’. As the highlights of Cambridge Analytica in 2015 and so-called Datagate conditions had been recently brought to global attention, the ‘disconnect’ option appeared as a political stance. Years into my missing out on the ‘social media’ experience, my position was even more emphasised by the popularisation of documentaries such as The Social Dilemma (2020).^5^

### Smart devices

The progressive disconnection that I embarked on was systematic but phased. After quitting all social media (Facebook, Instagram, Whatsapp), since 2018 I began to engage with my smartphone only as a sim-free tablet and used a GSM-only device to make calls.

My so-called ‘feature-phone’ lacked sensors – that normally in smartphones passively capture and calculate all sorts of metrics (altitude, barometers, GPS, accelerometer, pedometer, etc.), and was generally tougher built than normal devices.^6^ The plastic has heavier, and the screen is significantly less fragile. Internet navigation feature was exceptionally limited, yet somewhat functional. It could not offer any playback option, nor download any picture or file. When accessing email via the mini browser, I had to insert my email and password every single time, confirming that there appeared to be no cache or cookies. The navigation did not know my location, and I never fully grasped what my IP address was. In this odd grey area, I felt vaguely protected and anonymous. My device never featured any advertisement or requested Cookie consent. I expected the browser to simply accept cookies as default, but I am not sure whether this was the case.

### E-commerce and entertainment

Following the purpose of my offlife approach, I decided still in 2018 to unsubscribe from all those that I considered capta-gathering online services, including streaming providers of video/film and music media. Instead, I opted towards ‘things’ that offered per se a service that had no hidden, monitoring potential – similar to the more recent considerations of Han (2022) and his ‘non-things’. I began investing in hard-copy material (CD and DVD) or hard-drive-shared digital material that would allow me to enjoy music limiting the productions of capta (via clicks, algorithms, likes and suggestions). I would read a wide range of printed newspapers from libraries to receive updates not only on the ‘latest’ news, but also read about weather forecasts, shows and events. I also attempted to consult dedicated dictionaries to clarify meanings and translations, rather than websites. The same applied to other kinds of guides; I tried to reduce to the bare minimum my access to Wikipedia and other websites. My online purchases and transactions, given my disdain for use of data and working conditions of the gig economy and e-commerce providers, were reduced with great satisfaction to quasi-zero.

### Digital footprints

Navigation and directions without a GPS service were one of the most controversial features, meeting with the greatest resistance from friends and peers. In discussing directions, I oftentimes ended up seeking locations on somebody else’s map provider. Many peers sensed this as a ‘parasitic’ behaviour on my behalf: without my ‘capta’ of other users’ data, I could not find my whereabouts. I ‘compromised’ my commuting by using an offline portable Navigation device while driving, to which I manually updated the map via SD card. However, when on foot I would use an offline map app strictly disabling GPS.

Through these practices and recognitions, a slow but evident ‘skimming’ of my screen time was palpable. Through my capta disengagement, I came to feel that not only my data but also my attention was less ‘captured’, and thus stolen from me. To date, mixed arguments have been forwarded on the actual validity of digital detox programmes (Ellis, 2019; Ghita and Thorén, 2021) – a practice promoted both in mindfulness programmes and by academics. I did, however, by limiting card transactions, online banking, delivery services, and felt I was slowly regaining my lost-to-‘click-bait’ identity, formed by my stolen data (Zuboff, 2019). Such a process felt particularly liberating in regard to the typical ‘echo chamber’ information control and other ‘dark patterns’ in UX techniques, whereas users are subtly and covertly ‘tricked’ into ‘opting in’ to services rather than ‘out’ (Fard, 2022).

Within this context, my GSM-feature phone device did not seem powerless, quite the opposite. It appeared as a tool that neutralised the harm, to my persona and towards others. I could be trusted, as I had nothing to hide. Anyone could pick up my phone, unlock it and see my texts and calls. No sensible biometric data were stored, and no sensitive data were needed. In fact, my obliviousness to my phone’s whereabouts led me to lose it on repetitive occasions. The notoriously harmful perspective of the Fear of Missing Out (FOMO) syndrome resolved itself with the sudden realisation that there was not much that I was missing out on. Or better, that more could be achieved, with less. My feature device was an object that simply had no appeal and no value – nothing much to store and nothing much to hide. Less precious data, minor menacing capta. And by that, again increased considerably my sense of belonging to the people surrounding me and indifference to other more expensive, more problematic, more time-consuming and potentially harmful tools.

## ‘Dumbphone’ vs covidiots

With the outbreak of the Covid-19 pandemic in early 2020, the ‘successes’ of my offlife engagement (reduction of Internet interaction, screen time and online purchases), that I directly associated with captaveillance, became problematic. Since early 2020, in concomitance with the harshening of the virulence restrictions, I could no longer rely solely on newspapers, cash money, hard-copy entertainment or media in general, as I had done, with some difficulty and great satisfaction, until then. Emergency social isolation and restrictions made the digital world an inevitable portal to access and engage with all information, media, transport and transaction. In the name of health hazards, finding a digital compromise has clearly been a necessary price to the grounded ideas of digital resistance (Lavorgna et al., 2021). With the Covid Digital Certificate and contact-tracing solutions, the ‘smart device’ I addressed as ‘captivating’ device became a pivotal tool for hazard monitoring and social hygiene (Csernatoni, 2020). Big Data, with its problems, turned out to be essential to detect threats and potentially future outbreaks (Rathinam et al, 2021) – to the extend of conceiving a neologism of ‘coronoption’ (The Economist, 2020). In these terms, alternatives proved counterintuitive and were addressed by the socially stigmatising epithet of the ‘Covidiot’ (Trottier et al., 2021), embedded in the hate scrolling of those who did not abide by the newly established social rules. The feature phone that I was using, short of tracking features and sensors, became evidently a controversial and unjustified gizmo to strangers and institutional eyes. My innocent ‘dumb phone’ that was once started with curiosity, and at times pity, became transmogrified into a dangerous device of dissidence.

Nevertheless, the problematics that I traced in the replete scenario of onlife captaveillance have become ever more significant. Corporations and state agencies, through contact-tracing apps and the Covid Vaccine Certificates, proved extremely efficient and influential in using ‘captivating’ elements to control and ‘lower the curve’ of Covid infections (Sweeney, 2020). Contract tracing was used in practically all affluent societies (Floridi and Sotgiu, 2021). Contact-tracing apps have been promoted as a necessary citizen moral obligation for ‘everyone with a cell phone’ (Talesnik, 2021). Through these apps, the implication is that the more citizens comply, the better such tools function. Yet, beyond initial crash incidents (underlining that the formula ‘more downloads, better service’ was not really the case), these Apps have become central elements for the control and monitoring of the virus and its ‘carriers’ (Gasser et al., 2020). With Covid-19, the capta-dynamics have become pivotal in halting the virus: gathering health data via apps, but also offering immediate and highly effective surveillance practices of ‘public health surveillance, urban security, and workplace surveillance’ (Trottier et al., 2021: 109). The smartphone could trace the citizens, track their movements, but also allow the immediate and precise filming and reporting of the quarantine breachers, or general dissidents of the restrictions (The (New York Times, 2021). Resisting the captaveillance during the pandemic contributed to captaveillance itself, including episodes of moral vigilantism and denouncing morally corrupt citizens – reporting parties, crowded encounters, ‘out-of-perimeter’ or ‘family bubbles’ rendezvous to the authorities: episodes are easily recalled in countries across the globe. These took place in both active and passive episodes of captaveillance: citizens were passively monitored, and they actively monitored each other.

In Italy – one of the notoriously most casualised and restrictive European countries during the pandemic – Foreign Minister of the time Luigi Di Maio expressed dismay towards whoever was reluctant to use contact-tracing apps. Di Maio argued ‘ironically’ that corporations have been using our data for the past decades with hardly any user being worried (Fatto Quotidiano, 2020) – thus it was a natural predisposition to give up Data for the greater good. This rhetoric of privacy as a red herring during Covid-19 (Lyon, 2020) is reminiscent of the ‘privacy is dead’ claim made already 1999 by Sun Microsystems CEO Scott McNealy, who suggested users to ‘get over it’ (Sprenger, 1999). Such argument comes almost hand in hand with the recurring argument, ‘If you have done nothing wrong, you have nothing to hide’, which has not only been hardly criticised by the UK Biometric and Surveillance Camera Commissioner Fraser Sampson (2021) but proved quintessentially problematic during the pandemic – whereas the standards of what is wrong and what right may shift so suddenly and abruptly, and previous consent losing suddenly validity. However, this was not at the time nor should today be a justification for mindless techno-fatalism, denying ‘the importance of protecting personal data from unwanted surveillance or control from the government or big tech companies’ (Lavorgna et al., 2021: 37). eHealth Data may potentially turn into yet a further powerful currency of opaque capta functioning (Pasquale, 2015), as interestingly highlighted already in 2017 in an intriguing volume edited under the name ‘Under Observation’ (Adams et al., 2017).

Practices of resistance to these forms of surveillance practices, no matter how morally ambiguous and socially harmful in different circumstances, are still today in constant debate (Lavorgna et al., 2021). To resist a form of technological harm, or a ‘zemiosis’, in the words of Wood (2022), during the Covid-19 crisis has become, it seems, yet again an instance replicating unwanted harm. One had to choose the lesser evil (Williams and Dienes, 2021).

Indeed, the point of how ‘capta’ may be wrongfully gathered, especially so under exceptional circumstances, is well reported (see Kitchin, 2020; Lavorgna et al., 2021). Luciano Floridi himself, interviewed by Simona Sotciu (2021), noted not only the recurring dangers of the breaching and hacking of highly sensitive health data inefficiently stored by governments and health agencies, but also how these very governments have promoted strict technological discrimination towards whoever is not familiar or even is indisposed towards the technology. The digital health frontier, once a highly debated feature in guaranteeing a delicate balance between surveillance and discrimination (Lombardo and Buckeridge, 2007), within the m-health systems and ‘metaverse’ realities, are forming the norm for our future interaction in the onlife. For citizens and scholars, this reality turns into a further element of difficulty and indisposition: not only our privacy, but our sense of control faces recurrent and influential means of coercion and harm – social, corporate and institutional.

## The appearance of disappearance

For 5 years, I attempted a form of offlife friction that would loosen the ‘vigilant’ grip of the onlife. The suggested solution, promoted with my auto-ethnography, was to attempt an offlife subcultural existence, even if only partially. My aim was to experiment with ways of ‘life’ that did not produce endless content and ‘capta’ food. My solutions were mostly practical, mixing obfuscating software with physically self-limiting my digital engagement.

However, it became very quickly clear that smart devices, phones in particular, were particularly efficient in ‘capturing’ attention, not only data. Everything in it is designed to lock you to the screen – pick it up as often and as long as possible. While this sounds trivial, it becomes particularly impressive and truly demanding when attempting to ‘opt-out’.

It has been suggested that the digital scenario itself offers ideal conditions for a denial of responsibility, as a ‘result of forces beyond their control’ (Brewer et al., 2019: 5). My personal choice of no longer ‘giving’ data could have resulted in a micro-resistance in the system, proving that, partially, some of this may be possible. With Covid-19, captaveillance proved important and beneficial: it protected citizens by tracing their whereabouts, and it offered ‘safe and quick’ transactions through contactless payments. It has safely delivered groceries and goods. However, even before Covid-19, I noticed that my non-conformity was at best reluctantly tolerated by those fully engaged in the onlife scenario. With my choice of resisting captaveillance, what I seemed to be proposing was an alternative of voluntary ‘social isolation’, to which no user would abide: Deleuze’s assemblage takes over restlessly (Angwin, 2014), though with a purpose. As I have discussed, such oblivious control and permanent authentication are very well represented by smart devices, with our constant online and offline identification (see credit cards and the likes). Inspired by Agre, my conception of captaveillance comes to consider a further element: capturing as an effortless and lax practice. It is not a practice of control: it is a constant and incessant gathering of capta, and a unilateral one. It does not need coercion; it naturally and automatically engages in cohesion and dependence (Han, 2015).

Such consideration brings possibilities for further study, over the criminalisation of this kind of resistance – or indeed, specific instances and occurrences for the securitisation of data. This also requires further work on policy work, analysis and design of platforms and devices currently in use. In the time being, we can rely on platforms, software and add-ons that can scramble, obfuscate, limit and confuse our data – we can decide to opt out (semi) drastically, or struggle to maintain our captaveillance at bay. However, on a final note, once you play by the rules, going back is as hard as ever. Contactless payments are impressively swifter, simpler and by now socially accepted rather than cash. Clicking ‘accept’ on a website and when downloading an app is undeniably easier and more convenient than opting out – or not using it all. And convenience is often trivially associated with a progressive better, clearer and faster functioning for all of us. From this perspective, one can only slowly realise that although I was looking at corporate responsibility and the harms involved in capturing information, my research in ‘disappearing’ from the system ‘appeared’ instead ethically troubled. I was being inconsiderate to the dynamics of distress my approach generated towards others: it was them sensing my actions as problematic. My alternative did not prove a real alternative. Already before Covid-19, I had noticed how my offlife approach appeared unsensible and anachronistic. It seemed to create greater friction in the short term in societal values than in the technologies themselves. By the middle of the pandemic, participating in capta-dynamics was truly a necessary condition that allowed very few if any alternatives. Captaveillance, it would seem, by now is truly the norm.

## Conclusions: The cost of capta

It is uncertain how long GSM mobiles will be compatible with current antennas (Wasserstein, 2021), and whether the so-called feature phones will be accessible on the market – not to mention the use of ‘cash’, the non-conformity to social media, and the refusal to abide by professional metrics, digital citizenships and the likes. One would need to deploy even more tools, ‘obfuscation’ manoeuvres – forms of sousveillance and whatnot. To attempt to ‘disappear’ to this neo-normal system would seem to be even more controversial, impractical, and even anti-social. The pandemic has evidently proven so. As noted by *The Guardian* columnist Jen Wasserstein (2021), the ‘luxury’ of living without smartphone is since Covid-19 harder than ever. The number of smart devices has thrived, and concepts such as digital citizenship have achieved overwhelming significance in the government’s bureaucratic establishment. Also, capturing users’ whereabouts and encounters has been in fact effective in a moment of a health crisis.

However, this comes at a price. Other than the element of capturing even more information from users and citizens, the protection of information was accompanied with evident flaws (Floridi and Sotgiu, 2021), abuses and security breaches (Lyon, 2022); This becomes even more significant when the information recollected is triangulated, as with the Covid passports, among health, revenue, and immigration statuses (Gasser et al., 2020). Elements of harm and abuse become even more evident in the hands of the beholder, and we may perhaps indulge in the luxury of trust in the Global North, but not so much in other parts of the world. Since Covid-19, elements of captivating technologies again recall the Deleuzian aspects of control when we look at the exclusion, the marginalisation of those who are ‘outside’ the system (Calzada, 2022). Stateless citizens, liminal individuals, who may not, or do not, wish to be integrated into the capta-grinder become punished and further subjugated. Captaveillance returns with its targeting and insisting scenario (Wiener, 2020). Handing out information in this scenario is promoted not only as a lucrative exercise but as a civic duty. Cohesion yet again oppresses coercion. Resistance, it seems, becomes itself harmful. Desistance, deviant.

## Notes

1 I thank one of the reviewers of the article in framing so well this central distinction.

2 This point is inspired by Agre (1994) article, though the connection data vs capta is not present in the article.

3 One particularly helpful extension, AdNauseam, is promoted and developed by Helen Nissenbaum, offering a tool to ‘fight back unilateral surveillance’. Functioning is not simply an ‘add-block’ feature (limiting the view of an advertisement on a webpage), but scrambling the ‘real interest’ of the user by clicking literarily all advertisements contained in a webpage. Such a tool comes hand in hand with another related extension, TrackMeNot, that creates a ‘bot’ of random and incessant searches to scramble the user profile.

4
http://trackmenot.io/faq.html#random

5 My act was evidently perceived as a ‘hipster’ or ‘smug’ thing to do – basically an excuse for bringing attention to myself, or demonstrate some sort of ‘peculiarity’. Being without social media, especially Facebook, is not the centre of this article; it rather seemed as common sense to me at the time. The birth of my first daughter in 2018 reinforced the desire to increase my capta engagement. For the abovementioned reasons, now less than ever I wished corporations to know the details, store the pictures or speculate on this new existence.

6 The name of the model is not particularly important, as any ‘feature’ phone offer similar characteristics of the ones described.
